# Delayed diagnosis of vascular compression syndrome mimicking chronic gastritis: a case report

**DOI:** 10.1097/MS9.0000000000005052

**Published:** 2026-05-25

**Authors:** Abhaya Acharya, Sushant Shah, Lokmani Bhandari, Arpan Acharya, Sasisth Sah, Santosh Upreti

**Affiliations:** a Maharajgunj Medical Campus, Institute of Medicine, Tribhuvan University, Kathmandu, Nepal; b College of Medical Sciences, Kathmandu University, Dhulikhel, Nepal

**Keywords:** case report, epigastric pain, superior mesenteric artery syndrome, vascular compression, weight loss

## Abstract

**Introduction:**

The superior mesenteric artery (SMA) syndrome is a rare condition where the third part of the duodenum is compressed between the abdominal aorta and the SMA. It often causes nonspecific upper abdominal symptoms that mimic common problems like gastritis, leading to delays in diagnosis.

**Case presentation:**

A 23-year-old woman from central Nepal had 5 years of epigastric pain, nausea, occasional vomiting, acid brash, and gradual weight loss. She was repeatedly treated for gastritis without improvement. Initial ultrasound was normal, and endoscopy showed antral gastritis. Contrast-enhanced CT revealed a very narrow aorto–mesenteric angle (14°) and distance (3.6 mm), confirming SMA syndrome, along with the narrowing of the celiac trunk, thrombosis of the SMA, and signs of pelvic congestion. Conservative treatment failed, and she underwent open duodenojejunostomy. After surgery, her symptoms resolved completely, and she gradually gained weight.

**Discussion:**

This case shows that the SMA syndrome can be missed for years, especially when endoscopy shows only secondary gastritis. Early CT scanning and timely surgery can cure symptoms and prevent prolonged suffering.

**Conclusion:**

The SMA syndrome should be considered in young patients with chronic upper abdominal pain, persistent vomiting, and unexplained weight loss. Prompt imaging and surgical management are often needed for lasting recovery.

## Introduction

Superior mesenteric artery (SMA) syndrome is a rare cause of proximal small bowel obstruction and is associated with significant morbidity and mortality when diagnosis is delayed^[^1^]^. This condition occurs due to vascular compression, which reduces the aorto–mesenteric angle (typically <25°) and decreases the aorto–mesenteric distance (often <8–10 mm), resulting in partial or complete obstruction, proximal duodenal dilatation, and secondary gastric stasis^[^2^]^.

First described by von Rokitansky in 1842 and later detailed by Wilkie in 1927, the SMA syndrome has an estimated incidence of 0.013–0.3% on radiographic studies, though the true prevalence is likely higher due to underdiagnosis^[^3^]^. It predominantly affects young adults, especially females, and is frequently associated with rapid or significant weight loss, which depletes the mesenteric fat pad, narrows the aorto–mesenteric angle, and creates a cycle of malnutrition and worsening obstruction. Other predisposing factors include anatomical variants such as high insertion of the ligament of Treitz, spinal deformities, corrective scoliosis surgery, prolonged supine immobilization, severe burns, malignancies, and eating disorders^[^4^]^.

HIGHLIGHTS

- The superior mesenteric artery syndrome may masquerade as chronic gastritis, resulting in prolonged diagnostic delay.
- Persistent epigastric pain with unexplained weight loss should prompt the consideration of vascular compression syndromes.
- Contrast-enhanced computed tomography is essential for demonstrating reduced aorto–mesenteric angle and distance.
- Open duodenojejunostomy offers definitive and durable symptom relief in refractory cases.
- Early diagnosis and timely surgical intervention prevent prolonged morbidity.

Clinically, the SMA syndrome presents with nonspecific upper gastrointestinal symptoms, including postprandial epigastric pain, nausea, bilious or food-particle vomiting, early satiety, bloating, acid brash, and progressive weight loss^[^5^]^. These features often mimic more common disorders, such as chronic gastritis, functional dyspepsia, peptic ulcer disease, or gastroesophageal reflux, frequently leading to delayed diagnosis and prolonged conservative management with proton pump inhibitors or prokinetics^[^3^]^. Upper gastrointestinal endoscopy may reveal secondary antral gastritis or duodenitis due to stasis, further masking the underlying mechanical cause. Contrast-enhanced computed tomography (CECT) remains the gold standard for diagnosis, demonstrating the characteristic vascular compression and altered aorto–mesenteric measurements^[^6^]^.

Although many cases respond to conservative measures, including nutritional rehabilitation, positional therapy (e.g., left lateral decubitus or knee-chest position), and weight gain, refractory or chronic cases with significant weight loss and persistent obstruction require surgical intervention, most commonly duodenojejunostomy^[^7^]^.

We report the case of a 23-year-old female from central Nepal with a 5-year history of chronic epigastric pain mimicking refractory antral gastritis, progressive weight loss, and failure of medical therapy. Contrast-enhanced CT revealed SMA syndrome with reduced aorto–mesenteric angle and distance, celiac trunk narrowing, SMA thrombosis, and features of pelvic congestion syndrome. Surgical management with open duodenojejunostomy resulted in complete symptom resolution and sustained improvement. This case highlights the diagnostic challenges of the SMA syndrome, its ability to mimic common gastroduodenal disorders, and the importance of considering vascular compression in young patients with chronic, treatment-resistant upper abdominal symptoms and unexplained weight loss.

The case report has been prepared in accordance with CARE guidelines^[^8^]^.

## Case presentation

A 23-year-old female from central Nepal presented to a tertiary care hospital with a 5-year history of epigastric pain, which had progressively worsened over the preceding year. The pain was dull, aching, localized to the epigastrium, and more pronounced in the evening. It was associated with nausea, intermittent non-bilious vomiting containing food particles, and acid and water brash, without any signs of dehydration. She reported unintentional weight loss of approximately 8 kg over 1 year. There was no history of altered bowel or bladder habits, gastrointestinal bleeding, fever, shortness of breath, or other systemic symptoms.

On examination, she was hemodynamically stable. Abdominal examination revealed a soft abdomen with mild epigastric tenderness. No palpable masses or organomegaly were detected, and bowel sounds were normal.

**Figure 1.**
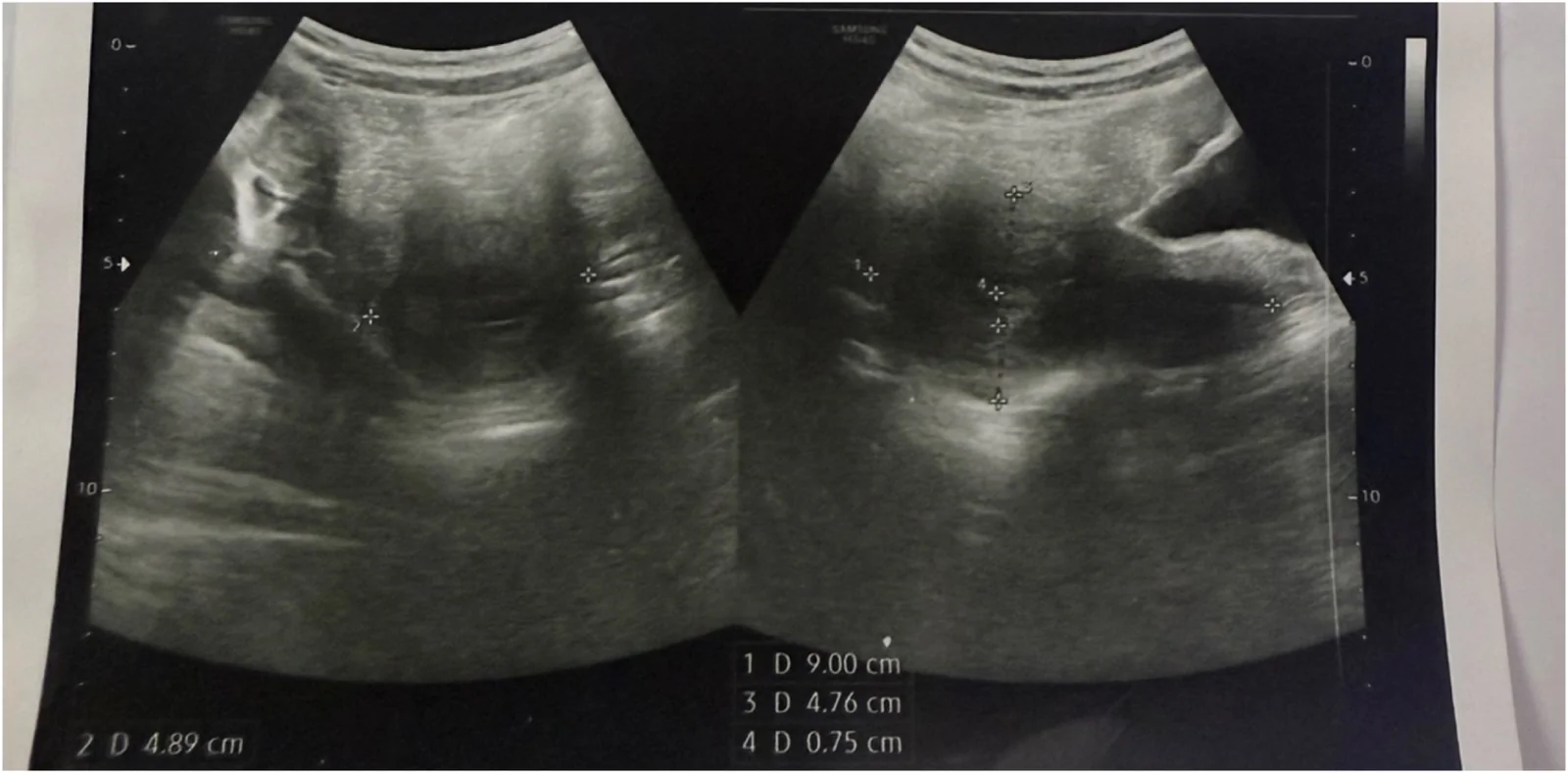
Ultrasonography (USG) of the abdomen and pelvis showing no abnormal findings.

**Figure 2.**
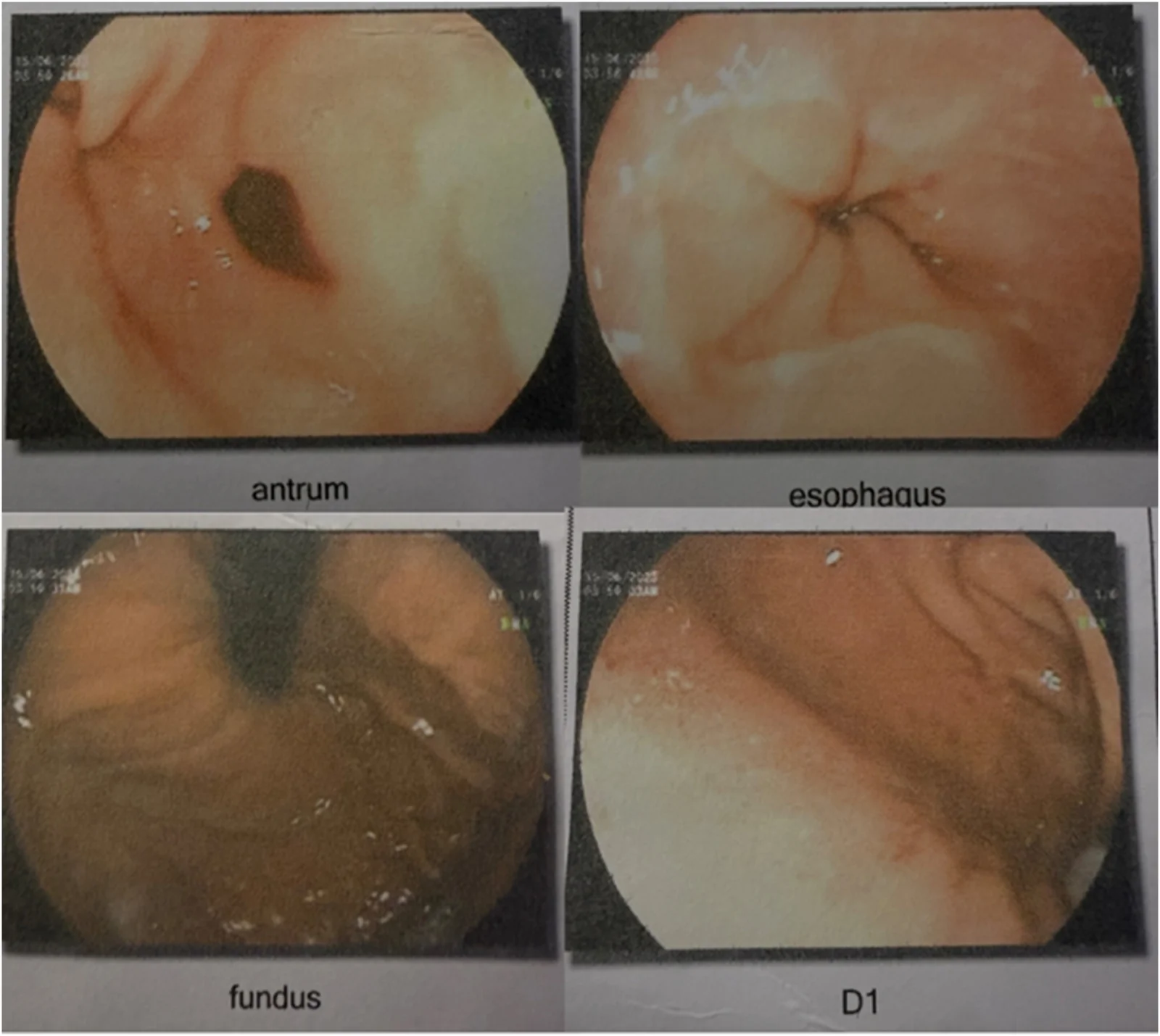
Esophago–gastro–duodenoscopy findings in a patient with abdominal pain. The esophagus, fundus, and body of the stomach appear normal. The gastric antrum shows patchy mucosal erythema, and the pylorus is erythematous, consistent with antral gastritis. The duodenum (D1 and D2) appears normal.

Initial laboratory investigations revealed a hemoglobin level of 9.8 g/dl, while other hematological and biochemical parameters were within normal limits. Abdominal ultrasonography (Fig. 1) was unremarkable. Upper gastrointestinal endoscopy (Fig. 2) demonstrated features of antral gastritis, and the patient received repeated medical management, which failed to improve her symptoms.

**Figure 3.**
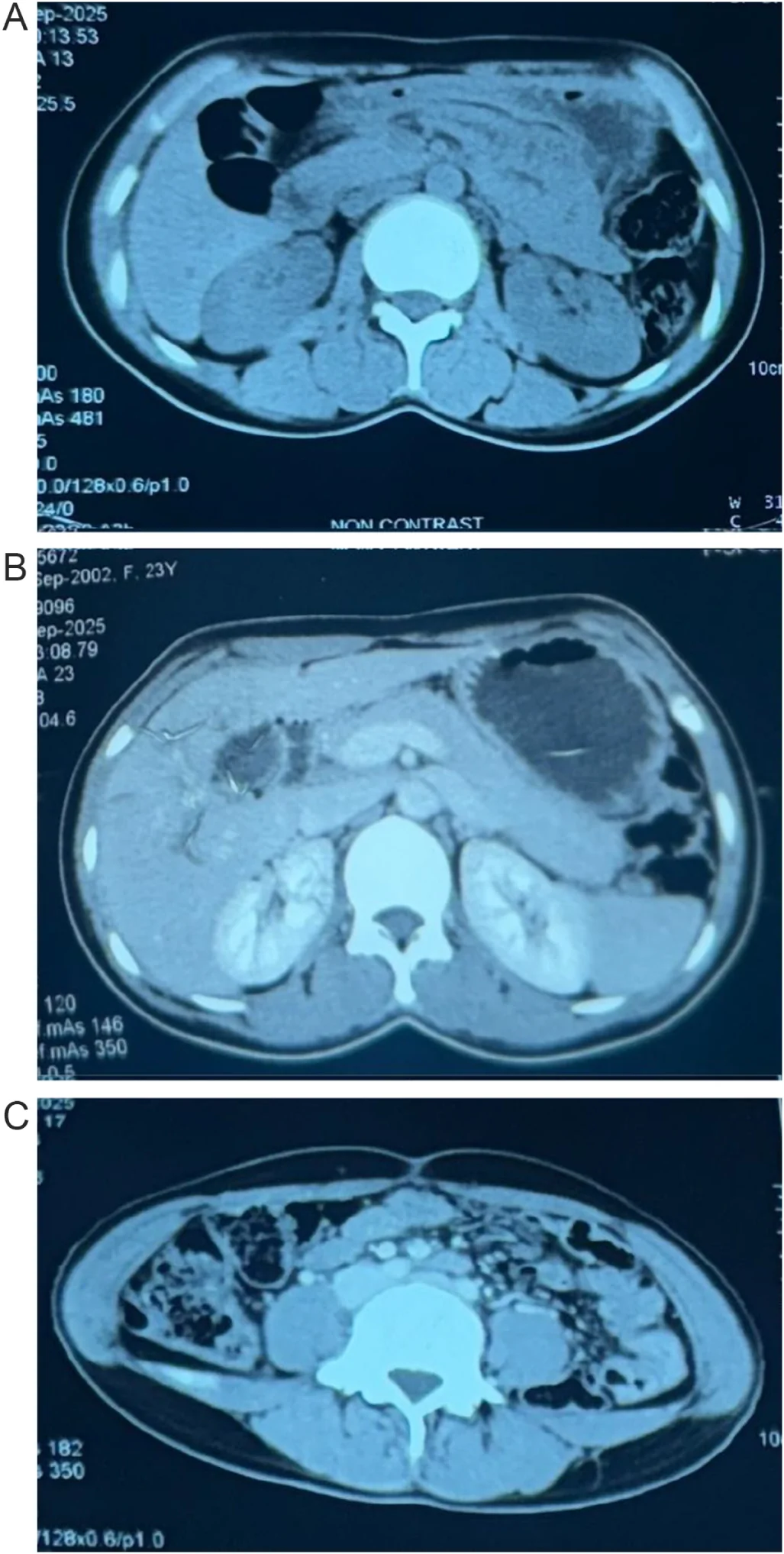
Axial CT images of the upper abdomen showing the superior mesenteric artery (SMA) syndrome. (A) Non-contrast CT demonstrates compression of the third part of the duodenum between the abdominal aorta (posterior) and SMA (anterior), with proximal dilatation of the stomach and first/second duodenal segments due to reduced aorto–mesenteric distance and fat pad depletion. (B and C) Contrast-enhanced CT (portal venous phase) highlights the aorta and SMA with duodenal narrowing at the compression site, marked proximal gastric and duodenal dilatation (fluid-filled), and possible concomitant celiac trunk narrowing/SMA thrombosis.

Given the chronicity of symptoms, weight loss, and poor response to conservative therapy, a CECT scan of the abdomen and pelvis was performed 2 months after the ultrasound. The scan revealed narrowing and dysfunction of the celiac trunk, SMA thrombosis, a reduced aorto–mesenteric angle (14°) and distance (3.6 mm), suggestive of aorto–mesenteric compression, and features consistent with the pelvic congestion syndrome (Fig. 3).

Due to persistent symptoms, radiological evidence of vascular compression, and failure of conservative management, surgical intervention was planned. An upper midline incision was made, and the first and second parts of the duodenum were mobilized via Kocherization. Retroperitoneal attachments were dissected to mobilize the third part of the duodenum, and the ligament of Treitz was divided to mobilize the fourth part of the duodenum and duodenojejunal flexure. Intraoperatively, mild dilatation of the first and second duodenal segments was noted. An adequate duodenal length was achieved, and the stomach anatomy and ligament of Treitz were otherwise normal.

A loop of jejunum approximately 20 cm distal to the duodenojejunal flexure was brought through a retrocolic window, and a 2-layer duodenojejunostomy was performed. The procedure was completed without intraoperative complications.

## Postoperative course and follow-up

The postoperative period was uneventful. The patient received standard postoperative care, including intravenous fluids, analgesia, and proton pump inhibitors. Oral intake was gradually initiated, progressing from clear liquids to a soft diet, which she tolerated well without vomiting or abdominal distension.

She reported significant improvement in epigastric pain during the hospital stay, and bowel function returned to normal. There were no postoperative complications, such as surgical site infection, anastomotic leak, or ileus. She was discharged in stable condition.

**Figure 4.**
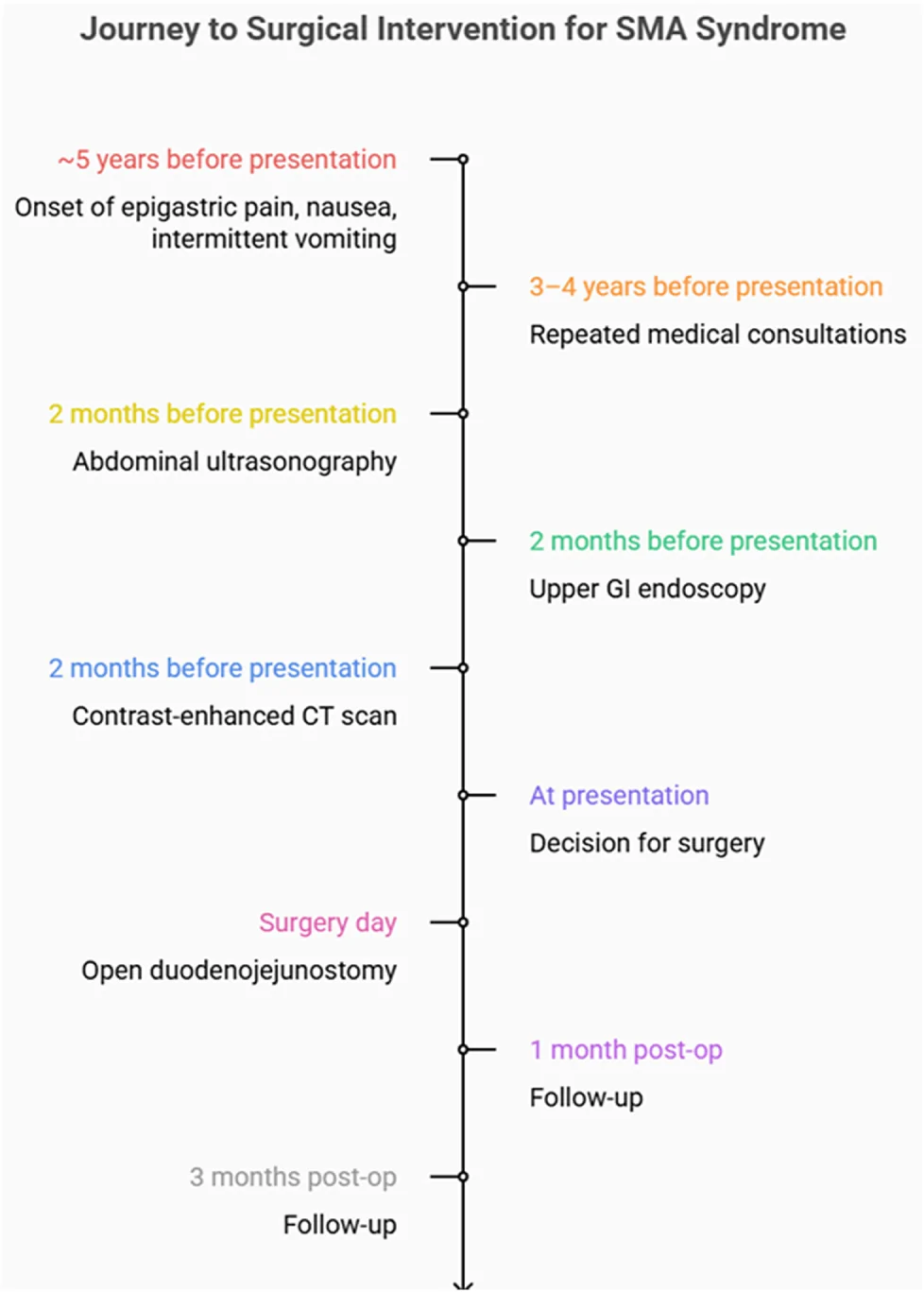
Timeline of the patient’s clinical course, investigations, diagnosis, and management of SMA syndrome.

At follow-up, the patient demonstrated sustained clinical improvement. At 1 month, she had complete resolution of vomiting and marked reduction of epigastric pain. At 3 months, she remained asymptomatic, with improved appetite and gradual weight gain. No recurrence of symptoms was noted during follow-up (Fig. 4).

## Discussion

The SMA syndrome is a rare but potentially serious cause of extrinsic duodenal obstruction, occurring when the third part of the duodenum is compressed between the SMA and the abdominal aorta^[^1^]^.

In this case, the patient had a 5-year history of epigastric pain, nausea, vomiting, and significant weight loss (8 kg in 1 year), illustrating the slow and often subtle onset of the SMA syndrome. Such presentations frequently mimic common gastrointestinal conditions like chronic gastritis or functional dyspepsia, which can delay diagnosis and perpetuate a cycle of malnutrition and worsening obstruction. The underlying pathophysiology is related to a reduced aorto–mesenteric angle (14°) and distance (3.6 mm, normal 10–28 mm), likely worsened by the depletion of the mesenteric fat pad due to weight loss. Laboratory findings, including anemia (hemoglobin 9.8 g/dl) and cachexia, further support chronic nutritional compromise. This cycle – obstruction leading to malnutrition, which in turn worsens vascular compression – highlights the importance of considering the SMA syndrome in young females with unexplained weight loss and refractory upper gastrointestinal symptoms.

The diagnostic delay in this case, spanning several years, was largely due to misattribution of symptoms to antral gastritis on endoscopy. Endoscopic findings often reflect secondary changes such as gastritis or duodenitis from proximal stasis rather than the primary mechanical obstruction^[^5^]^. Abdominal ultrasonography, although widely available and non-invasive, was non-diagnostic as it does not reliably assess vascular angles in subtle compressions. In contrast, CECT proved to be essential, by confirming the reduced aorto–mesenteric measurements and revealing associated anomalies including celiac trunk narrowing with dysfunction, SMA thrombosis, and pelvic congestion syndrome^[^5,9^]^. The coexistence of these vascular compressions is uncommon; celiac involvement may suggest overlap with a median arcuate ligament syndrome, whereas SMA thrombosis may reflect thrombotic complications of chronic stasis, increasing the risk of ischemia^[^4^]^. A pelvic congestion syndrome added diagnostic complexity but did not dominate the clinical presentation. This multifactorial vascular involvement underscores the importance of comprehensive imaging for planning management in complex cases.

Management of this patient with conservative measures, including nutritional support, positional therapy (e.g., left lateral decubitus), and proton pump inhibitors, was unsuccessful, consistent with the reports showing that advanced cases with persistent obstruction and severe weight loss often require surgical intervention^[^7^]^. The patient underwent an open, 2-layer retrocolic duodenojejunostomy, using a jejunal loop 20 cm distal to the duodenojejunal flexure, without intraoperative complications^[^1,7^]^. Intraoperative findings of mild proximal duodenal dilatation confirmed chronic obstruction without ischemia, allowing safe mobilization via Kocherization, retroperitoneal dissection, and division of the ligament of Treitz^[^10^]^. Alternative procedures, such as gastrojejunostomy or Strong’s procedure, were not performed due to higher recurrence or complications in chronic compression with thrombosis. Laparoscopic approaches, although increasingly favored, were not feasible in this tertiary center due to resource constraints and surgical expertise.

The postoperative course was uneventful, with rapid resumption of oral intake, resolution of vomiting and epigastric pain, and sustained weight gain by 3 months, reflecting outcomes reported in surgical series where early mobilization and perioperative care mitigate complications such as anastomotic leak or ileus^[^9^]^. The absence of symptom recurrence aligns with long-term success rates for duodenojejunostomy exceeding 85%^[^3^]^.

Limitations of this report include its single-patient nature, limiting generalizability. Long-term follow-up beyond 3 months was not available. Histopathological correlation and postoperative vascular imaging were not performed. The relationship between the SMA syndrome and associated vascular anomalies (celiac narrowing, SMA thrombosis, and pelvic congestion) cannot be definitively established, warranting further research.

## Conclusion

This case highlights the diagnostic challenges of the SMA syndrome, particularly in the presence of multifocal vascular compression and nonspecific endoscopic findings. An early consideration of the SMA syndrome in young patients with chronic, treatment-resistant upper gastrointestinal symptoms and unexplained weight loss is essential. Contrast-enhanced CT is invaluable for diagnosis and surgical planning. In refractory cases, open duodenojejunostomy can provide durable symptom relief and break the malnutrition-obstruction cycle. Future research should explore endovascular or minimally invasive approaches for associated vascular anomalies to improve multidisciplinary management in complex presentations.

## Data Availability

The data that support the findings of this study are available from the corresponding author upon reasonable request.
